# Surface Design for Antibacterial Materials: From Fundamentals to Advanced Strategies

**DOI:** 10.1002/advs.202100368

**Published:** 2021-08-05

**Authors:** Wenlong Li, Eng San Thian, Miao Wang, Zuyong Wang, Lei Ren

**Affiliations:** ^1^ Department of Biomaterials State Key Lab of Physical Chemistry of Solid Surface College of Materials Xiamen University Xiamen 361005 P. R. China; ^2^ Department of Mechanical Engineering National University of Singapore Singapore 117576 Singapore; ^3^ College of Materials Science and Engineering Hunan University Changsha 410082 P. R. China

**Keywords:** antibacterial materials, antibiofilm, bacterial targeting, clinical infection, surface modification

## Abstract

Healthcare‐acquired infections as well as increasing antimicrobial resistance have become an urgent global challenge, thus smart alternative solutions are needed to tackle bacterial infections. Antibacterial materials in biomedical applications and hospital hygiene have attracted great interest, in particular, the emergence of surface design strategies offer an effective alternative to antibiotics, thereby preventing the possible development of bacterial resistance. In this review, recent progress on advanced surface modifications to prevent bacterial infections are addressed comprehensively, starting with the key factors against bacterial adhesion, followed by varying strategies that can inhibit biofilm formation effectively. Furthermore, “super antibacterial systems” through pre‐treatment defense and targeted bactericidal system, are proposed with increasing evidence of clinical potential. Finally, the advantages and future challenges of surface strategies to resist healthcare‐associated infections are discussed, with promising prospects of developing novel antimicrobial materials.

## Graphical abstract

This review provides a systematic overview of recent progress on advanced surface modifications to prevent bacterial infections, starting with the key factors against bacterial adhesion, followed by varying strategies that can inhibit biofilm formation effectively. Finally, the advantages and future challenges of surface strategies to resist healthcare‐associated infections are discussed, with promising prospects of developing novel antimicrobial materials.

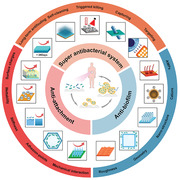

## 1 Introduction

Bacteria, widely found in human living and working environments, is one type of the oldest specie on the earth.^[^ ^1^ ^]^ While most of the bacteria are harmless to human health, bacteria such as *Staphylococcus aureus, Helicobacter pylori, Escherichia coli*, *and Bacillus pestis* can invade the host and cause various infectious diseases including pneumonia, endocarditis, septicemia, and osteomyelitis.^[^ ^2^ ^]^ Nowadays, healthcare‐acquired infections pose a major threat to human health, which may cause even serious complications to the patients, leading to heavy burdens for society.^[^ ^3^ ^]^ Traditional methods that prevent clinical infections include applicational aseptic technology and systemic antibiotics therapy, but the latter does not often have a very satisfactory outcome.^[^ ^4^ ^]^ For instance, at the time of contamination on medical devices ^[^ ^5^ ^]^ (e.g., catheters, artificial prosthetics, subcutaneous sensors, and orthopedic implants), systemic antibiotic therapy is reported to have an effective success rate of only 22–37%.^[^ ^6^ ^]^ In addition, for a specific infected site, systemic administration of antibiotics usually requires a high dose, which may induce cytotoxicity and side effects to surrounding tissues.^[^ ^7^ ^]^ Moreover, this strategy can lead to bacterial drug resistance.^[^ ^8^ ^]^ It is reported that drug‐resistant bacteria could claim ten million lives a year, and by 2050, this number will surpass the deaths due to cancer.^[^ ^9^ ^]^ To better treat the infections caused by drug‐resistant bacteria, various antimicrobial drugs ^[^ ^10^ ^]^ (e.g., antibacterial peptides and amphiphiles) and antimicrobial materials ^[^ ^11^ ^]^ (e.g., nanoparticles, hydrogels, engineered surfaces, and surface coatings) have been developed. However, the situation of bacterial resistance has not been alleviated effectively. According to the UK‐commissioned report, the economic loss caused by drug‐resistant bacteria is still increasing year by year.^[^ ^9b^ ^]^ How to eliminate bacteria effectively without causing bacterial resistance has become an important research nowadays.

Recent developments greatly enrich our knowledge that the material's surface plays a decisive role in bacterial infection processes.^[^ ^12^ ^]^ Bacteria can be adsorbed onto the material's surface under actions including Coulomb force, Van‐der‐Waals force, or H‐bond. The bacteria then aggregate, degenerate flagella, and begin to secrete extracellular matrix (ECM) to achieve irreversible adhesion to the surface.^[^ ^13^ ^]^ Therefore, it is reasonable to develop effective strategies interfering with the interactions between the bacteria and the material's surface,^[^ ^14^ ^]^ which can be achieved by altering the morphology, hardness, roughness, and chemical constituents, and in turn, hindering the communication between bacteria and finally, delaying biofilm formation.^[^ ^15^ ^]^ During the past decades, the number of publications related to the terms “antimicrobial surfaces”, “implantable device infection” and “biofilm” is increasing continuously. Surface modification for the development of new antibacterial materials has attracted much attention from a wide scope of researchers.^[^ ^15^, ^16^ ^]^

**Figure 1:**
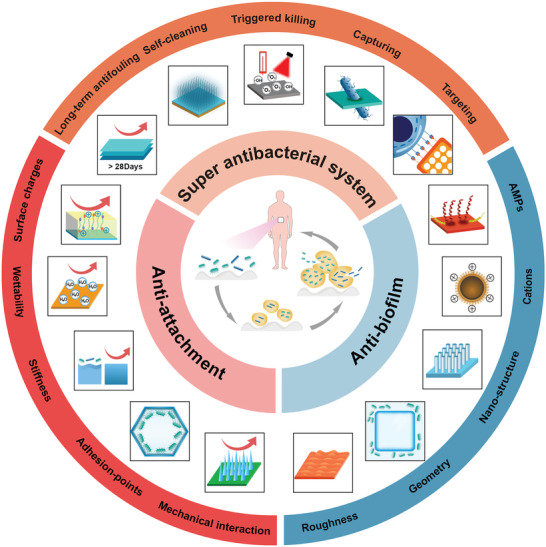
Overview of surface design for antibacterial materials. Left: the key factors (surface charge, wettability, stiffness, adhesion‐points, and mechanical interaction) on material surfaces against bacterial adhesion. Right: various effects (roughness, geometry, nanostructure, cations, and AMPs) to resist biofilm. Upper: super antibacterial system for clinical therapy through various strategies (long‐term antifouling, smart self‐cleaning, triggered killing, bacterial capturing, and bacterial targeting).

In this review, we provide a comprehensive review on the research progress and clinical potential of antibacterial surface‐modification strategies (**Figure** **1**). This report will include three parts: 1) surface modifications such as stiffness, wettability, surface charge, and surface morphology for preventing bacterial adhesion; 2) surface modifications such as patterning and germination for antibiofilm; and 3) advanced strategies for resisting clinical infections. Bacteria may contaminate the surfaces of medical devices and implants, causing wound infections, bacteremia, tissue inflammation, and implant infections. Due to the severity of the clinical infection, multifunctional abilities of antimicrobial materials are required such as high‐efficiency antibacterial effects, long‐term effects, biocompatibility, and bacterial targeting. This report thus enlightens selective excellent antibacterial strategies in recent years and offers a perspective on surface modification strategies for the proper design of future antimicrobial materials.

## 2 Progress in Surface Modifications for Preventing Bacterial Adhesion

Bacterial contamination and its related infections pose a severe threat to hospitals, food industry, and community settings.^[^ ^17^ ^]^ Although antibiotics and sterile conditions are widely used, there are still a large number of patients suffering from biomaterial‐related infections, thus resulting in biomedical devices' failure. We have used the terms “adhesion” and “attachment” to describe the different stages about the interaction between bacteria and material surface. “Adhesion” describes a physicochemical process comprising of bacterial reversible and irreversible adhesion. The term “attachment” represents that bacteria have deposited on material surface successfully and begin to secrete lipopolysaccharide.^[^ ^18^ ^]^

**Figure 2:**
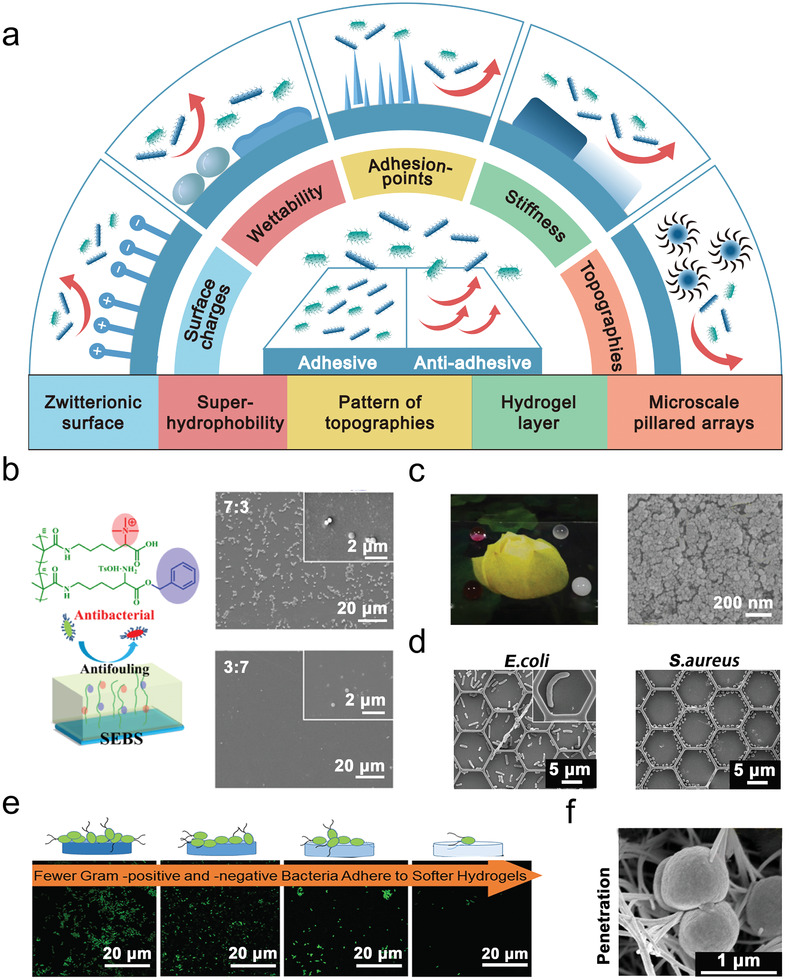
Surface modifications for preventing bacterial adhesion. a) Key surface factors (surface charge, wettability, adhesion‐points, stiffness, and topographies) against bacterial adhesion. b) Surface charge. A zwitterionic surface of LysAAm‐QAC and LysAAm‐OBzl (feeding ratio of 3:7) against bacterial adhesion. Reproduced with permission.^[^ ^20a^ ^]^ Copyright 2019, American Chemical Society. c) Wettability. An F‐CuO‐SiO_2_ superhydrophobic surface with extremely low bacterial adhesion. Reproduced with permission.^[^ ^20b^ ^]^ Copyright 2018, American Chemical Society. d) Adhesion points. *E.coli* and *S. aureus* with random distribution and predominant adhesion along the side‐walls respectively in the 5 µm honeycomb‐like patterns. Reproduced with permission.^[^ ^20c^ ^]^ Copyright 2015, Elsevier B.V. e) Stiffness. PEGDMA‐Agar‐coated surfaces with decreasing stiffness (from 6489 to 44.05 kPa) attached by fewer bacteria. Reproduced with permission.^[^ ^16a^ ^]^ Copyright 2015, American Chemical Society. f) Topography. Ti surface with microscale pillared arrays for preventing bacterial adhesion by mechanical penetration. Reproduced with permission.^[^ ^20d^ ^]^ Copyright 2018, Springer Nature.

Bacterial adhesion triggers the onset of infection on the biomaterial's surface and is a primary targeting step for developing antibacterial strategies. Generally speaking, bacteria prefer to grow on surfaces rather than in aqueous environments via non‐specific interactions, such as hydrogen bonding, electrostatic forces, hydrophobic interactions, and Van‐der‐Waals force. These interactions may be attractive or repulsive, depending on the complex interaction between the bacteria and the surface.^[^ ^19^ ^]^ If the intrinsic characteristics of bacteria are excluded, adhesion behaviors on a surface are strongly determined by the surface's chemical properties (e.g., surface charges and hydrophobicity/hydrophilicity) and topologies (e.g., surface roughness, geometry, and other physical configurations).^[^ ^16^, ^20^ ^]^ The understanding of these factors will certainly unveil the underlying mechanisms for anti‐adhesive surface (**Figure** **2**a).

### 2.1 Surface Charges

Surface charge is a very important property that has a decisive effect on the interaction between materials and bacteria. In general, bacterial cells are coated with a peptidoglycan layer (consisting of sugars and amino acids), which decides that most bacteria are negatively charged. Therefore, the positively‐charged material surface is widely used to capture or kill bacteria whilst the negatively‐charged material surfaces can repel bacteria of polyanionic glycosides (mostly Gram‐positive bacteria) through electrostatic interaction. However, this repulsion depends largely on the species of bacteria. For instance, the Gram‐positive bacteria with polycationic glycoenzymes can adhere to the negatively‐charged surface more easily than Gram‐negative bacteria with polyanionic glycocalyx.^[^ ^16b^ ^]^ Interestingly, Ishihara et al.^[^ ^21^ ^]^ first reported a zwitterionic surface bearing both cationic and anionic groups. This zwitterionic surface could resist protein absorption and subsequently bacterial adhesion. With further research, the anti‐adhesion mechanism of this zwitterionic surface has been revealed:^[^ ^22^ ^]^ 1) the zwitterionic surfaces with strongly ionic solvation can bind water molecules strongly and stably through electrostatic interaction, which can form a hydration layer (an energetic and physical barrier) to resistant protein adsorption; 2) the neutrality‐charged zwitterionic surface can minimize the electrostatic interaction between protein/bacteria and material. Following the mechanism, different families of the zwitterionic materials with high hydrophilicity have been developed and proven to possess anti‐adhesion capability.^[^ ^23^ ^]^ For example, Lv et al.^[^ ^20a^ ^]^ modified a silicon wafer with a zwitterionic surface composed of hydrophilic lysine methacrylamide (LysAAm), positively‐charged quaternary ammonium (QAC), and hydrophobic benzyl (OBzl) groups. This hydrophilic and neutrality‐charged surface could obtain a high anti‐adhesion performance to bacteria (bacterial coverage < 2%) (Figure 2b).

### 2.2 Surface Wettability

Wettability, one of the most momentous features of a solid surface, can affect a variety of processes related to bacterial infections such as adsorption, wetting, adhesion, friction, and lubrication.^[^ ^24^ ^]^ A moderate wettable surface can easily enable bacterial adhesion through hydrogen bonding and hydrophobic interactions between peptidoglycan and surface, respectively.^[^ ^25^ ^]^ Interestingly, superhydrophilicity, and superhydrophobicity can inhibit bacteria approaching the surfaces, thereby resisting bacterial adhesion.^[^ ^26^ ^]^ The term superhydrophilicity can be rooted back to 1996, which represents a water contact angle on surfaces lower than 5^o^.^[^ ^27^ ^]^ The first super hydrophilic coating that consisted of a thin TiO_2_ polycrystalline film was reported by Wang et al.,^[^ ^28^ ^]^ with a contact angle of nearly 1^o^. In 1992, Bergström et al.^[^ ^29^ ^]^ reported a PEG‐coated polystyrene surface to reduce fibrinogen adsorption. From then onwards, PEG^[^ ^30^ ^]^ has been adopted in a wide range of antibacterial adhesion surfaces. For example, Qi et al.^[^ ^31^ ^]^ grafted PEG brushes on Ti surface which achieved a good anti‐adhesion effect within 24 h. To further improve the anti‐adhesion ability of the superhydrophilic surface, bactericidal substances were introduced into the PEG coating. For example, Yang et al.^[^ ^32^ ^]^ developed an anti‐adhesion coating comprising of dopamine (for adhesion of the polymer to a substrate), cations (for bacterial killing), and PEG (for anti‐adhesion property). This coating was able to retain its anti‐adhesion activities for over 14 days in bacterial suspension (10^5^ CFU mL^–1^). Moreover, a combination of topographical structures is an effective strategy to enhance anti‐adhesion ability. For example, close‐packed hexagonal arrays of nanocones developed on silk films were able to enhance surface hydrophilicity significantly, to repel bacteria adhesion by more than 90% (Gram‐negative and Gram‐positive).^[^ ^33^ ^]^ A proposed mechanism underlying the hydrophilic nanocones to cause bacterial death could be explained by the repulsion of bacterial cell wall, which is induced by the increased pressure due to a significant reduction in the bacterial‐surface contact area.

Inspired by the self‐cleaning properties of lotus leaves, Barthlott, and Neinhuis proposed the concept of superhydrophobic surfaces.^[^ ^34^ ^]^ Some oxide nanoparticles (e.g., SiO_2_, CuO, and TiO_2_)^[^ ^35^ ^]^ and some polymers (e.g., polydimethylsiloxane, polypyrrole, and polyvinylidene fluoride)^[^ ^36^ ^]^ with low surface energy, have been employed to produce superhydrophobic surfaces, which can trap a stable air layer and, therefore, reduce the contact area between the material and the liquid to prevent bacterial adhesion. For example, a transparent superhydrophobic surface with multiscale roughness, prepared by spraying of silica sol and CuO nanoparticles on a glass substrate (F‐CuO‐SiO_2_), could obtain the extremely high bacterial anti‐adhesion rate (99.9%) and sterilization properties (Figure 2c).^[^ ^20b^ ^]^ However, design with a superhydrophobic surface alone cannot achieve complete elimination of bacterial adhesion due to the disappearance of superhydrophobic status when the concentration of bacteria exceeds a threshold value.^[^ ^37^ ^]^ An effective strategy to solve the problem is to develop a switchable superwettable surface. For example, Deˇkanovský et al.^[^ ^38^ ^]^ reported a superhydrophobic and switchable material surface based on a highly stretchable silicon polymer doped with polypyrrole. In general, this surface underwater could form an air gap. When bacterial adhesion occurs, it switched to a hydrophilic state under an electric field, further releasing the drugs (crystal violet) to kill bacteria in time and keeping surface clean.

### 2.3 Available Contact‐Adhesion Points

Bacteria adhere preferentially to irregularities in order to maximize the bacteria‐surface area. This is dependent on the sizes and shapes of the bacteria relative to those surface topographies. For example, scratches in an order of bacterial size can increase the contact area between bacteria and the material's surface, whereas the binding potential of bacteria on structures larger or wider than bacterial size, tends to approach that of a flat surface. Specifically, the surface's topographies determine bacterial preferential adhesion points, through which bacteria can differentiate upper and lower topographies and choose the positions to settle on the surfaces (Figure 2d).^[^ ^20c^ ^]^ The principles behind these observations are associated with an adhesion point theory,^[^ ^39^ ^]^ and when the organisms are smaller than the feature size of characteristic surfaces, bacteria can achieve a large available contact area, shield the surface roughness, and obtain higher adhesion strength. Complementary to this, a different theory shows that the stiffness of the bacteria cell wall can limit bacterial free‐adaption to topographical features at very small sizes,^[^ ^40^ ^]^ which is supported by findings in which bacteria tend to adhere, preferably at square corners and convex features rather than on flat or concave walls.

Surface irregularities are characterized effectively in terms of roughness that has various expressions (e.g., Ra, an average surface roughness value; Rq, root‐mean‐square roughness; and Rpv, peak to valley roughness). The effects of these surface parameters on bacterial adhesion are well demonstrated, by a significant reduction in bacterial adhesion (at least ten folds lower) and single‐bacteria distribution on rough surfaces (rather than microcolonies clusters on polished surface topography).^[^ ^41^ ^]^ In another study, Rajab et al.^[^ ^42^ ^]^ suggested that roughness, together with other topographical parameters such as feature geometry, chemistry, and physicochemistry, was able to decrease bacterial adhesion. For example, in combination with superhydrophobicity, the material surface can obtain optimized anti‐adhesion ability. Similarly, the combination between different surface topographies (e.g., laser‐induced periodic surfaces of ridges/grooves and nanopillars) and low surface wettability can endow material surface with better capability of shielding bacterial adhesion points.^[^ ^43^ ^]^

In addition, the engineering of surface structure has been proved as an effective way to obtain excellent anti‐adhesion results. Recently, to uncover how surface topography interferes with bacterial adhesion, a wide range of engineered‐design structural features have corroborated a significant impact on bacterial adhesion for reproducible antibacterial performances. Among these, engineered surfaces with nanoscale roughness, micrometer lateral dimensions, and well‐defined features of different sizes and shapes can have different degrees of reduction in bacterial adhesion, through the control of the initial cell location.^[^ ^40^ ^]^ These selective sites for bacteria adhesion and limited bacteria number are also presented by different researchers,^[^ ^44^ ^]^ although the underlying mechanisms remain unclear. Yang et al.^[^ ^20c^ ^]^ suggested a contact‐based effect involving energetically favorable adhesion sites and physical confinement, which contributed to the excellent anti‐adhesion of engineered surface topographies (e.g., honeycomb‐like structures). In agreement with this, Ge et al.^[^ ^45^ ^]^ proposed a combination between the limited contact area availability and spatial confinement size‐effect using pillar patterns for effective antibacterial ability (e.g., 62% reduction in *S. aureus* adhesion, and 73% reduction in *E. coli* adhesion). Importantly, they demonstrated no lethal effects to bacteria on the as‐fabricated surface topographies.

Preferential adhesion points can also influence bacteria motility, thereby interfering with bacterial adhesion. It is well demonstrated that bacteria tend to align perpendicularly to line patterns and attach more randomly at higher densities when the patterns get wider to approach to flat surfaces.^[^ ^46^ ^]^ This bacterial organization has a close relation with longer cell‐body morphology and higher transcriptional activities of flagellar genes, thereby changing cell rotation patterns and settling preference. Based on this principle, surface topographies with hexagonal patterns could suppress the adhesion and colonization of *E. coli* and *S. aureus* remarkably.^[^ ^20c^ ^]^

Similar to a surface that offers preferential adhesion points, topographies with antibacterial chemistry can be designed for enlarged exposure of the anti‐adhesive points, and thereby increased antibacterial efficiency. For example, due to an increase in bacteria‐material contacts, surfaces with MgO micro rods could have faster bacterial elimination than commercially available MgO.^[^ ^47^ ^]^ As such, implant coating with laser‐induced conformal 3D porous structures was found to interfere with bacterial shape, and in turn, their adhesion and proliferation.^[^ ^48^ ^]^ This inhibited bacterial adhesion relies greatly on bacterial‐surface contact, suggesting that a surface with hierarchical topographies could deliver more anti‐adhesive and/or initial killing effects on bacteria, as evidenced by a nanoimprinted PLLA/Si, Sr‐nHA surface.^[^ ^49^ ^]^

### 2.4 Surface Stiffness

Materials stiffness, an easily tunable property of the surface, represents a different strategy for effective reduction in bacterial adhesion. This may offer synergistic effects with the traditional anti‐adhesion strategies via structure‐property relationships. Due to structural dissimilarity, both flagellated gram‐negative *E. coli* and non‐motile gram‐positive *S. aureus* behave differently to various substrate stiffness. This explains a bacterial behavior to prefer stiffer surfaces with increased adhesion. However, insights on how the surface stiffness interferes with the initial adhesion of bacteria remain unclear. It has been suggested that bacteria adhesive to the stiffer surface via both chemical interactions (e.g., Van der Waals, electrostatic and hydrophobic forces) and mechanical clues (e.g., roughness, patterns).^[^ ^50^ ^]^ Complementary to this, in a recent study by Kolewe et al.,^[^ ^16a^ ^]^ bacterial adhesion was found to be less dependent on material chemistry. During the cell‐surface interaction, bacteria tended to attach more on poly(ethylene glycol) dimethacrylate (PEGDMA)‐Agar‐coated surfaces with increasing stiffness from soft (44.05–308.5 kPa), to intermediate (1495–2877 kPa), and too stiff (5152–6489 kPa) (Figure 2e).

However, the pathogenic infections on ultrasoft biomaterials (<≈100 kPa) may rely more on the mechanical interaction via viscoelasticity. These lower elastic modulus makes the surface to be more compatible with delicate tissue structures. In this paradigm, Wang et al.^[^ ^51^ ^]^ demonstrated a different observation that *S. aureus* displayed decreased adhesion rate on stiff surfaces, with reduced numbers of adherent bacteria on the surfaces with increased modulus. This correlation between stiffness and bacterial adhesion possibly depends on bacterial (e.g., shape and motility), material and experimental condition.^[^ ^51^ ^]^ The bacteria‐surface interactions follow a thermodynamic principle with reversible bacterial adhesion, of which a layer of highly viscous adjacent water facilitates bacteria adhesion to the surface, minimizes the interfacial free energy, and in turn, increases the reversibility of interaction between the biological entities and the interface.^[^ ^51^ ^]^

### 2.5 Surface Mechano‐Bactericidal Interactions

Bacterial deposition onto a surface topography may place a significant stretching strain on the bacterial cell membrane. This mechanical effect will lead to bacterial membrane rupture if the stretching force is greater than the elasticity of the cell membrane, causing bacteria lysis and death. Such “deformation‐stretching‐torn apart effect” is mostly applicable to surface topographies comprising of pillar structures and has been theorized regardless of material surface chemistry.^[^ ^52^ ^]^ This killing effect at the initial stage of bacteria‐surface interactions can be accounted for the inhibition in bacterial adhesion and be manipulated through the design of the pillar's physical characteristics (e.g., radii, height, and density). For example, a two‐tier microscale pillared arrays demonstrated a decline in bacteria adhesion via mechanical rupture on the cell membranes.^[^ ^52^ ^]^ Similarly, this mechanical rupture effect was observed in nanoscale features (e.g., pocket‐type fibers), to penetrate and compress the bacteria in‐between the network of intertwined nanospheres (Figure 2f).^[^ ^20d^ ^]^ Surprisingly, recent observations also suggested that a syngenetic bactericidal effect of the protruding features, as evidenced from a multiscale roughness that comprised of nano and microstructures accounting for highly biocide‐free bactericidal property.^[^ ^53^ ^]^

Moreover, this biocide‐free mechanical effect can exhibit varying anti‐adhesion ability by different surface designs. For example, replication of the nanopillar structures with parameters comparable to those on the surface of cicada wing showed only a minor and negligible biocidal effect on the Gram‐positive pathogen of *S. aureus*.^[^ ^54^ ^]^ In contrast, it obtained nearly 100% bactericidal efficiency to *S. aureus*, with a surface topography comprising of pillars at a density of ≈40 pillars mm^–2^ and surface roughness of 39.1 nm. Structural densities >70 or <20 pillars mm^–2^, with surface roughness less than 20 nm, would result in no obvious bactericidal efficiency.^[^ ^54^ ^]^ Another study indicated that a decrease in pillar height, pillar cap diameter, and inter‐pillar spacing might be more effective in reducing bacterial adhesion for both Gram‐positive bacteria (e.g., *S.aureus*) and Gram‐negative bacteria (e.g., *P. aeruginosa*).^[^ ^55^ ^]^

## 3 Progress in Surface Modifications for Anti‐Biofilm

**Figure 3:**
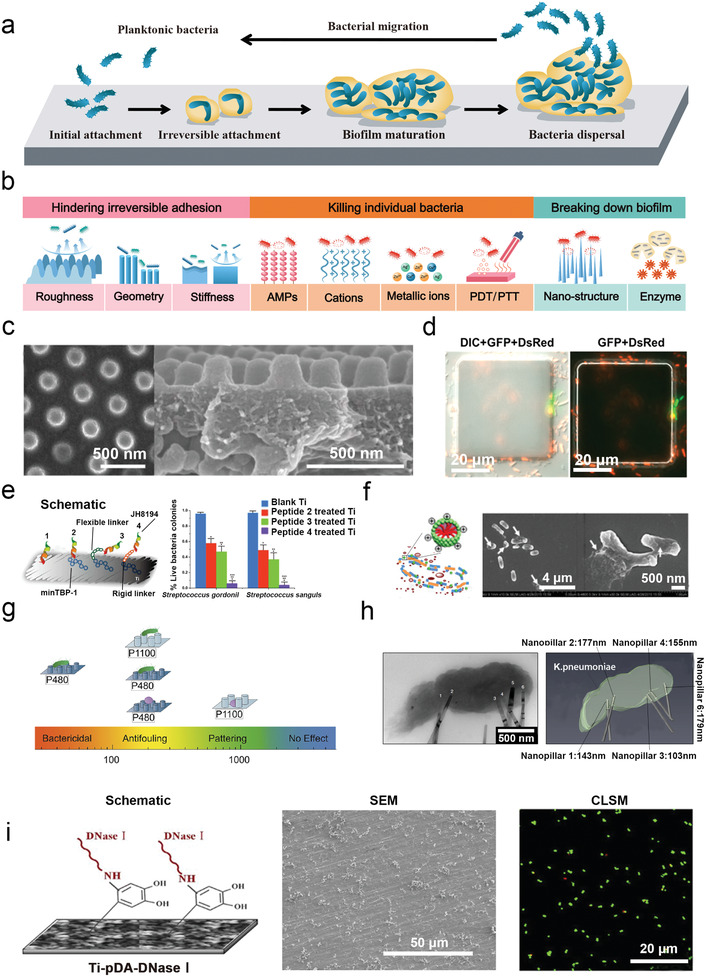
Surface modifications for anti‐biofilm. a) The process of bacterial biofilm formation. b) Various surface strategies for antibacterial biofilm. c,d) Strategy of hindering irreversible attachment. c) TiO_2_ nanoscale pillar with antifouling ability, and topography‐induced effects in hindering bacterial irreversible attachment. Reproduced with permission.^[^ ^16d^ ^]^ Copyright 2016, American Chemical Society. d) PDMS surfaces with 50 µm × 50 µm face‐up patterns for hindering bacterial irreversible attachment. Reproduced with permission.^[^ ^16e^ ^]^ Copyright 2017, American Chemical Society. e–h) Strategy of killing individual bacteria. e) AMPs (JH8194 peptide) on TiO_2_ surface with different linkers minTBP‐1, minTBP‐1‐flexible linker, and minTBP‐1‐rigid linker pose diverse bactericidal effects of 10%, 40%, 60%, and 90% respectively. Reproduced with permission.^[^ ^56^ ^]^ Copyright 2016, American Chemical Society. f) Cationic PTAC nanoparticles kill individual bacteria by bacterial membrane disruption. Reproduced with permission.^[^ ^57^ ^]^ Copyright 2016, American Chemical Society. g) PDMS nanopillar arrays (pitches of 1100 and 480 nm) with antibacterial effects (bactericidal, antifouling, and pattering) against the microbe at larger (*E. coli*) and smaller (*S. aureus*) sizes, respectively. Reproduced with permission.^[^ ^16f^ ^]^ Copyright 2020, American Chemical Society. h) Nature‐mimicked TiO_2_ nanopillars to kill bacteria by penetrating the bacterial envelope at least 100 nm. Reproduced with permission.^[^ ^16g^ ^]^ Copyright 2020, Springer Nature. i) Strategy of breaking down biofilm. The pDA‐DNase I coating on the titanium surface to hydrolyze eDNA of bacteria for biofilm breakdown. Reproduced with permission.^[^ ^58^ ^]^ Copyright 2018, Springer Nature.

Most of the anti‐adhesion surfaces cannot eliminate completely bacterial deposition, which leads to biofilm formation through the accumulation of biomolecules, cells, and organisms. The deposition of biofilm thus results in high costs and endangers human lives across many endeavors, including maritime operations, medicine, food industries, and biotechnology. Biofilm formation starts with direct contact between planktonic bacteria and surfaces. Then bacterial biofilm develops circularly through four stages including: 1) initial adhesion, 2) irreversible attachment, 3) biofilm maturation, 4) bacteria dispersal, and 5) bacterial migration (**Figure** **3**a).^[^ ^13^ ^]^ During this process, biofilm formation can be strongly influenced by the physicochemical property of the material's surface. Recently, surface modification has shown excellent performance in hindering irreversible attachment, killing individual bacteria, and breaking down the biofilm directly (Figure 3b).

### 3.1 Hindering Irreversible Attachment

The transition from reversible adhesion to irreversible attachment is the first step in the formation of biofilm on the surface.^[^ ^13^ ^]^ In order to delay such processes, material surfaces using proper topographical design are promising for hindering biofilm growth. For example, Choi et al.^[^ ^16d^ ^]^ developed a TiO_2_ nanoscale pillar, which endowed the TiO_2_ surface with excellent topography‐induced effects in modulating both organic protein and bacterial foulants. This topographical effect that depended largely on the relative size of the foulants, had an enhancement on both the overall and local shear stress to interfere with bacterial proliferation (Figure 3c). In addition, this synergetic effect of hydrophilicity and anti‐biofouling could be obtained via a replication of the biophotonic nanostructures, inspired by the rough topography found on the wings of the butterfly.^[^ ^59^ ^]^ To improve the antibacterial ability of the strategy, Imani et al.^[^ ^60^ ^]^ reported a rough topography combined with liquid perfluorocarbons can obtain the broad liquid repellency, further leading to reduced biofilm formation even after contacting with bacterially contaminated surfaces.

Interestingly, surface topography can be designed to change the stability of biofilm to reduce irreversible attachment for developing selective antifouling effects on certain species.^[^ ^61^ ^]^ Studies on surface topographies (e.g., laser texturing) have demonstrated the performance to reduce biofilm formation and total volume of biomass production from various bacteria, including *S. aureus*, *P. aeruginosa*, and *P. gingivalis*.^[^ ^62^ ^]^ In agreement with this, the organized micro‐topographic surface patterns at nanoscale roughness are also able to reduce biofilm formation on biomedical devices (22–58% for *Genus Staphylococcus*).^[^ ^40^ ^]^ Recently, Singh et al.^[^ ^48^ ^]^ confirmed that the inhibition in biofouling associated with laser‐textured surfaces was independent of chemical hydrophilicity. These observations validated the material's anti‐biofouling performance raised from surface topography.

Surface topography to modulate biofilm formation and biofouling may rely greatly on its structural parameters such as surface stiffness and geometry. Wang et al.^[^ ^51^ ^]^ demonstrated that a decrease in the interfacial coverage of biofilm was related to the increased modulus on soft surface topographies (Young's modulus of <100 kPa), suggesting a possible interferent effect on biofilm formation. More complex interactions between bacteria and the geometries may impact the biofouling process. For instance, with consistency in surface chemistry, Kargar et al.^[^ ^63^ ^]^ demonstrated a delayed biofilm formation on spheres with larger diameters (e.g., 1500 nm) than smaller ones (e.g., 450 nm), which showed the effect of surface curvature on bacteria behavior. Moreover, they proposed that the enlarged spacing between favorable cell sites could hinder early‐stage biofilm formation. In contrast, Mok et al. reported that a concave periodic boundary geometry tended to decrease the average bacterial concentration (>50%) at its boundary to a flat surface.^[^ ^64^ ^]^ These findings may have helped in exploring how the local variety in boundary curvature influences the bacterial biofouling process (e.g., scattering and accumulation dynamics).

Biofilm surface coverage is related to the initial number of adhered bacteria and the geometric height/depth of surface topography. In this paradigm, bacterial growth on surface topography resulted in decreased cell cluster formation (versus flat surface: 14‐fold).^[^ ^46^ ^]^ Complementary to this, Gu et al.^[^ ^16e^ ^]^ explored another ““hot spot”” that was stronger for bacterial conjugation than the parameters of the structural top and bottom did. The hot spots were located at the vertical sides of the topographic features, and together with the inter‐pattern distance, which can be used for hindering irreversible attachment in different ways. For example, a 10 *μ*m‐tall protruding pattern of poly(dimethylsiloxane) (PDMS) surfaces with an inter‐pattern distance equal to or >10 µm promoted biofilm formation, whilst a side length of 15 µm and inter‐pattern distance of 2 µm could reduce biofilm formation. The hot spots to disturb cell expansion and proliferation were suggested through physical confinement and structural barrier, which might have close relations to the bacterial motility (Figure 3d).

Theoretically, Chang et al.^[^ ^65^ ^]^ explored bacterial behaviors on a modeled topographical steps and found that bacteria had a reduced probability of traversing the solid‐liquid interface across steps when the step heights were similar to the cell's diameter (e.g., >0.9 µm), a time penalty for crossing the steps when their heights were similar to bacterial length (e.g., 2–3 µm), and instead of traversing the steps, a reorientation response of bacterial cell body when the step height was even higher. Watson et al.^[^ ^66^ ^]^ further established a simple surface energy model that explained the physical action of bacteria (deformation/rupture) on protruding patterns (e.g., nanopillars). In addition, the surface morphology of the material can avoid irreversible attachment by changing the activity of planktonic bacteria. For example, the height of surface micropillars interferes with the movement of water droplets, resulting in the formation of octagonal or square deposition patterns.^[^ ^67^ ^]^ This might change the local cell distribution and lead to elevated bacterial viability of the planktonic cells, with low biomass deposition.

### 3.2 Killing Individual Bacteria

The reproduction and aggregation of bacteria on the material surface are critical for biofilm formation.^[^ ^13^ ^]^ Therefore, killing individual bacteria is the most common and effective way against biofilms. Bactericidal surfaces can be modified effectively with various antimicrobial substances such as antibiotics, metal ions, antimicrobial peptides (AMPs), cationic molecules, and antimicrobial enzymes. Among them, antibiotics are not recommended due to their disadvantages of increasing bacterial resistance.^[^ ^68^ ^]^

Metal ions (e.g., Ag^+^, Zn^2+^, and Cu^2+^) are regarded as an effective antibacterial agent because of their strong broad‐spectrum antibacterial activity and low drug resistance. The antibacterial mechanisms of these metal ions are divided into three types: 1) metal ions that can enter bacterial cells through ion channels and trigger the Fenton reaction to produce excessive reactive oxygen species, to increase the permeability of the bacterial membrane and oxidative stress in the bacterial cells;^[^ ^69^ ^]^ 2) metal ions that can be transferred into toxic secondary metabolites, to affect the metabolic activities of bacteria;^[^ ^70^ ^]^ 3) excess heavy metal ions that can induce changes in bacterial genetic information (e.g., 16S rDNA).^[^ ^71^ ^]^ Following these mechanisms, metal ions are widely used in the surface modification of materials to kill bacteria. For example, Zhao et. al.^[^ ^72^ ^]^ introduced Mg and Ag on titanium surfaces by a plasma immersion ion implantation technique. This design endowed the titanium surface with an excellent bactericidal effect against *E. coli* (93%) and *S. aureus* (97%). In addition, the combination of metallic ions with surface topography can also show surprising antibacterial effects. For instance, Cheng et al.^[^ ^73^ ^]^ modified a titanium surface with metals (Sr and Ag) and nanotubes through an anodized and hydrothermal treatment. By adjusting the number of released metal ions, they realized a good bactericidal ability of the modified titanium implant and excellent osteogenesis for the new bone formation within four weeks. Similarly, Zhang et al.^[^ ^74^ ^]^ introduced Cu into the nanopores of nickel‐titanium implant to form an antibacterial surface coating. The work showed that the incorporation of Cu endowed the implants with high bactericidal ratio (93%). Meanwhile, the modified nickel–titanium implant realized improved cytocompatibility and promoted the proliferation of bone marrow mesenchymal stem cell.

AMPs have been considered to be a kind of promising candidate for replacing conventional antibiotics, due to their multiple targets and non‐specific antibacterial mechanism. The antibacterial mechanism of AMPs is extremely complex. It can be broadly divided into two categories:^[^ ^75^ ^]^ 1) AMPs that aggregate on the surface of bacterial cell membrane under electrostatic interaction and ion replacement, and then undergo conformational changes and vertically inserted into the cell membrane to result in different forms of membrane damage; 2) AMPs that enter the bacteria to interfere with the synthesis of DNA and RNA and to inhibit the activity of enzymes, leading to the death of bacteria. AMPs exhibit excellent anti‐biofilm activity against multi‐drug resistance and also isolate bacterial biofilms clinically, which can be attributed to the intervention in the early biofouling stage and/or the destruction of mature biofilms via promoting microorganism's separation and killing.^[^ ^76^ ^]^ For example, Liu et al.^[^ ^77^ ^]^ reported a composite mesh coated with an antibacterial gel containing AMPs. This strategy enabled sustain release of AMPs (RRRGRRRGPPGRRRGRRR, 10 mg cm^–2^) for over 10 days and resist *S. aureus* or *E. coli*. To further improve the anti‐biofilm effect of AMPs, Liu et al.^[^ ^56^ ^]^ studied the binding behavior of JH8194 AMPs on the Ti surface and demonstrated that AMPs with different linkers (minTBP‐1, minTBP‐1‐flexible linker, and minTBP‐1‐rigid linker) exhibited diverse antibacterial effects of 10%, 40%, 60%, and 90% respectively (Figure 3e).

Cationic molecules (e.g., quaternary ammonium salt) can kill bacteria effectively based on the contacted‐killing effect.^[^ ^78^ ^]^ Namely, the cationic molecules can firstly be adsorbed to the bacterial cell wall via electrostatic action, and then diffuse to the bacterial to disturb the membrane's potential, leading to membrane damage, cytoplasm leaking, and bacterial death.^[^ ^79^ ^]^ However, cationic molecules are often associated with high charge‐related toxicity.^[^ ^78^ ^]^ Therefore, the amphiphilic property is introduced in cationic molecules to reduce cytotoxicity and enhance the selectivity to the bacterial membrane.^[^ ^80^ ^]^ For example, Zhou et al.^[^ ^57^ ^]^ developed a cationic nanoparticle covered by a star‐shaped cationic tetrameric surfactant to kill *E. coli* efficiently, with a very low minimum inhibitory concentration (0.93–1.70 µm). Moreover, those nanoparticles exhibited insignificant cytotoxicity because the bacterial surface is more negatively charged than the mammalian cell surface (Figure 3f).

In addition, rising temperature on the surface can also cause irreversible damage to bacterial biofilm.^[^ ^81^ ^]^ For example, heating processes such as pasteurization, have been applied for antibacterial activities before the discovery of Penicillin.^[^ ^82^ ^]^ It is believed that common bacteria could be disinfected at a temperature above 55 °C due to the denaturalization of the heat shock proteins.^[^ ^82^ ^]^ In particular, near‐infrared (NIR) photothermal therapy (PTT) has gained popularity and is very quickly developed in recent years due to high light‐thermal conversion efficiency.^[^ ^83^ ^]^ PTT relies on the photo‐absorbers to generate heat from light absorption to burn biofilm. The unique light‐to‐heat conversion property of some nanomaterials (carbon‐based nanocomposites,^[^ ^83^ ^]^ noble metallic nanomaterials,^[^ ^84^ ^]^ metallic compound composites,^[^ ^85^ ^]^ and polydopamine^[^ ^86^ ^]^) have been utilized to produce novel and effective therapeutics bacterial killing. For example, using electrostatic surface self‐assembly technique, Yang et al.^[^ ^87^ ^]^ used negatively charged polystyrensulfonate and positively charged 3‐aminopropyltrimethoxysilane as the coupling agents to deposit Au nanorods on a piranha‐treated Ti surface, killing more than 60% of bacteria within 20 min.

Reactive oxygen species (ROS)‐mediated photodynamic therapy (PDT) as another new modality against biofilm,^[^ ^88^ ^]^ involves the combination of light with a photosensitizing drug in an oxygen‐rich environment.^[^ ^89^ ^]^ The production of ROS can be divided into two types^[^ ^90^ ^]^ :1) charge is transferred to the photosensitizer resulting in the generation of superoxide radicals (O_2_^–^·) which can be converted to hydrogen peroxide (H_2_O_2_) and free hydroxyl radicals (HO·); 2) energy is directly transferred to molecular oxygen (^3^O_2_), which can result in producing singlet oxygen (^1^O_2_). ROS are highly reactive and toxic, leading to bacteria damage and death through the oxidization of nucleic acids, proteins, nucleic acids, and lipids.^[^ ^91^ ^]^ For instance, Zhao et al.^[^ ^92^ ^]^ reported a photodynamic antibacterial nanoparticle prepared by the covalently conjugation between chlorin e6 photosensitizer and poly(allylamine hydrochloride)‐coated silica nanoparticles. This nanosystem can cause the noticeable damage to bacteria at low concentrations (166 µg mL^−1^). In addition, the photosensitizer could be loaded into hydrogels and modified to the material surface.^[^ ^93^ ^]^ Yao et al. loaded a photosensitizer methylene blue on the cyclodextrin‐modified hyaluronic acid through a host‐guest interaction, and prepared a photodynamic antibacterial hydrogel coating on material surface by the combination of ultrasonic spraying and photo‐crosslinking techniques.^[^ ^93^ ^]^ This coating could kill 99% of MRSA under light irradiation (660 nm, 30 J cm^−2^).

However, the excessive heating (above 50 °C^[^ ^94^ ^]^) and ROS (beyond the capacity of the cell's antioxidant systems ^[^ ^95^ ^]^) can lead to the damage to normal tissue through the hyperthermia/oxidation‐induced denaturation of cell proteins and irreversible cell destruction.^[^ ^96^ ^]^ For example, to improve the bactericidal effect of PTT at an appropriate temperature, Yu et al.^[^ ^97^ ^]^ reported a polydopamine‐coated Fe_3_O_4_ nanoparticle loaded with NO donors. This nanoparticle had synergistic photothermal and NO antibacterial effects, which at a low temperature (45 °C) could kill 80% of bacteria (*E. coli* and *S. aureus*) growth within 5 min. Similarly, Huang et al.^[^ ^98^ ^]^ reported a platform with special surface topography (silicon nanowire arrays) that was coated by photosensitive protoporphyrin IX and *β*‐CD‐mannose_7_ (a bacterial specific binding molecule) to improve the bactericidal efficiency and biocompatibility of ROS. Compared with a flat surface, such surface morphology could increase the material's contact area with bacteria and specifically capture the bacteria by *β*‐CD‐mannose_7_, allowing ROS to kill the bacteria at a high efficiency approaching to 96.7%.

Some wings of cicada species have been confirmed to pose species‐dependent bactericidal effects.^[^ ^99^ ^]^ Inspired by this, some special surface morphology has been designed to realize bacteria‐killing effects. For example, Shahali et al.^[^ ^100^ ^]^ developed surface topographies mimicking the different wings of three species (*Psaltoda Claripennis, Aleeta curvicosta*, and *Palapsalta eyrei*), and found a significant reduction in the colonies of *P. aeruginosa* and *S. aureus* after 18 and 2–4 h, respectively. This suggests that the killing effect of surface topography may depend on the characteristics of both structure and bacteria. To explore how topographical geometric parameters influence surface bactericidal naturally, Xue et al.^[^ ^101^ ^]^ mentioned that killing bacteria via surface topography was dependent on the relative structure‐cell rigidity. Experimentally, observations on a nanoengineering Ti surface by Cao et al.^[^ ^20d^ ^]^ demonstrated the relationship between delayed biofilm formation and structure geometries of a pear‐type and pocket‐type nanospheres. In agreement with this, Heckmann et al.^[^ ^16f^ ^]^ further uncovered the above structure‐property relationship. Pitches of 1100 and 480 mm have an antifouling effect on a larger microbe of *E. coli* and a smaller microbe *S. aureus*, respectively. PDMS nanopillar arrays with pitches of 1100 and 480 nm have antifouling effects against the microbe at larger (*E. coli*) and smaller (*S. aureus*) sizes, respectively. For certain nanopillar arrays (e.g., 480 nm), the surface structure could exhibit antibacterial properties against *E. coli*, whilst antifouling effect against *S. aureus* (Figure 3g).^[^ ^16g^ ^]^ In addition to this, instead of causing rupture or lysing the bacteria, an oxidative‐stress‐related killing mechanism was proposed by Jenkins et al.,^[^ ^16g^ ^]^ by which nature‐mimicked TiO_2_ nanopillars could deform the shape of Gram‐positive and Gram‐negative bacteria and penetrate their envelope, to inhibit bacterial division and trigger the production of reactive oxygen species with an increased abundance of oxidative stress proteins (Figure 3h).

Recent developments further explore the roles of the synergistic bactericidal effects of surface chemistry and morphology. For example, a wrinkled graphene oxide surface geometry with sharp ridges/grooves demonstrated a time‐dependent effect on interfering bacterial viability, with nearly complete killing efficiency of ≈99% being achieved.^[^ ^102^ ^]^ This was attributed to the wrinkled surfaces that could trap diameter‐matched bacteria for enhanced cell‐surface interaction and promote effective charge transfer at the sharp edges. This led to the oxidation of the bacterial components, with substantial damages to the bacterial membrane followed by leaking of the intracellular substances and, finally, bacterial death. Similarly, thin layers of Ag and/or Cu coated on a tall (8–9 mm high) and sharp (35–110 nm) nanostructured surface could kill bacteria (efficiency of ≈97%) within 30 min.^[^ ^103^ ^]^ In contrast, ZnO nanopillar coatings on zinc foil and steel surfaces displayed a bacterial killing efficacy several orders higher than the ZnO nanopillars coated on other surfaces.^[^ ^104^ ^]^ This phenomenon was attributed to the combination of topographical cell‐wall rupture and superoxide released from the ZnO coating, with electrons donated from the galvanized interface. However, in these strategies, submicrometer‐sized roughness was suggested to outweigh the surface chemistry, in delaying biofilm formation (Gram‐negative and positive bacterial strains) over a long period of exposure time (>7 days).^[^ ^105^ ^]^ In addition, the topography can also be combined with the ROS generated from PDT to kill bacteria in the dark and light. For instance, Xu et al. reported an Ag_3_PO_4_ decorated urchin‐like TiO_2_ nanoparticle.^[^ ^106^ ^]^ The urchin‐like topography on the surface of nanoparticles could physically puncture bacterial cells from all directions and combined the effect of ROS and Ag^+^ releasing for a synergistic antibacterial efficiency. After light irradiation for 20 min followed by darkness for 12 h, the strategy allowed for high antibacterial efficacy of 99.76 ± 0.15% and 99.85 ± 0.09% against *E. coli* and *S. aureus*, respectively.

### 3.3 Breaking Down Biofilm

The biofilm matrix mainly contains a variety of biological macromolecules such as extracellular polysaccharide, extracellular DNA (eDNA), enzymes, and other proteins.^[^ ^107^ ^]^ Therefore, the destruction or elimination of biofilm‐related components is meaningful for biofilm breakdown.^[^ ^108^ ^]^ For example, eDNA plays an important role in stabilizing bio0film structure and bacterial resistance,^[^ ^109^ ^]^ and studies have shown that deoxyribonuclease I (DNase I) can hydrolyze effectively the eDNA of Gram‐positive bacteria and eukaryotic microorganisms, destroying the stability of young biofilms.^[^ ^110^ ^]^ Recently, Ye et al.^[^ ^58^ ^]^ used dopamine as an intermediate to modify DNase I (pDA‐DNase I) on Ti surface, forming a highly hydrophilic DNase I coating, which showed a significant effect in preventing the adhesion and biofilm formation of *S. aureus* within 24 h, and favorable biocompatibility by cell study in vitro (Figure 3i). In addition, disturbing the quorum sensing of bacteria can also be an effective anti‐biofilm strategy.^[^ ^111^ ^]^ Quorum sensing is a special mechanism existing among bacteria, which involves the life activities of the bacterial community, such as the adjustment of bacterial density and biofilm formation.^[^ ^112^ ^]^ For example, the peptide modification to disturb the quorum sensing of bacteria can inhibit biofilm formation. Accordingly, Kim et al.^[^ ^111^ ^]^ modified the autoinducer peptide (AIP‐I) onto material surface, resulting in the dispersal of *S.aureus* biofilm and realized an ≈80% reduction in the surface biofilm coverage.

Surface renewal can occur when the adhesion energy is larger than an effective bending stiffness on a surface.^[^ ^113^ ^]^ This property can be used to block links between bacteria, thus further breaking down the biofilm. This effective bending stiffness relates directly to the elasticity and thickness of the fouling patch, as well as the depth of the boundary layer of the wrinkled topography. Inspired by the natural surfaces that may be excellent in self‐renewal and preventing biofouling, Pocivavsek et al.^[^ ^113^ ^]^ proposed a biophysics‐based mechanism for surface renewal, of which a critical surface curvature was determined by an intrinsic characteristic length scale of the fouling layer.

Another strategy for breaking down mature biofilm is based on the shape memory polymers (SMP) with defined surface topography.^[^ ^114^ ^]^ In this paradigm, the surfaces can have rapid shape changes upon stimuli to remove established biofilms and thus, offer a more prolonged antifouling property. This strategy is applicable to various bacteria. For example, the total reduction rate of *P. aeruginosa* biofilms can be 99.9% as compared to the static flat control. A further study carried out by Lee et al.^[^ ^115^ ^]^ reported the bacterial killing efficiency in the presence of 50 µg mL^–1^ tobramycin could be more than three logs (2479‐fold) when compared to the static flat control, because the disruption of biofilm structure by SMP‐based dynamic surface topography could make bacteria become sensitive to antibacterial drugs.

## 4 Super‐Antibacterial Systems

**Figure 4:**
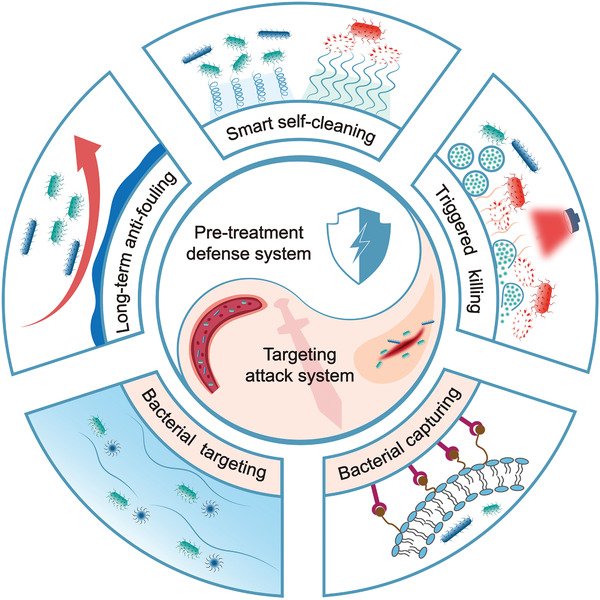
The super antibacterial systems. The pre‐treatment defense system to protect of implants and medical devices via long‐term antifouling, smart self‐cleaning, and triggered killing. The targeted bactericidal system to capture bacteria in wounds and deep organs via electrostatic attraction, receptor‐ligand interactions, covalent bonding, bacterial trapping bionics modification, and micro/nanorobots.

Clinical infections (e.g., wound infections, bacteremia, tissue inflammation, and implant infections) bring a big challenge to the maintenance of long‐term effectiveness, efficiency, durability, and safety of the antimicrobial materials in the medical system.^[^ ^116^ ^]^ Many efforts on surface functionalization (e.g., antifouling,^[^ ^117^ ^]^ contact‐killing,^[^ ^118^ ^]^ and biocide‐releasing surfaces^[^ ^119^ ^]^) have been done in order to avoid bacterial infections.^[^ ^120^ ^]^ However, existing strategies suffer from three major drawbacks, including short lifetime, toxicity, and antimicrobial resistance.^[^ ^121^ ^]^ Recently, researchers noted the problems and developed various promising surface modification strategies from the perspective of practical applications^[^ ^16^, ^122^ ^]^ (**Figure** **4**). All these works can be broadly divided into two categories: 1) pre‐treatment defense system: surface pre‐treatment can endow medical instruments with the ability of antibacterial adhesion or bacterial killing, to avoid bacterial infection during therapy. This is a type of passive defense strategy to protect medical instruments; and 2) targeted bactericidal system: targeted modification endows bactericidal materials with the ability to track bacteria in wounds, blood, tissues, and implants. This strategy not only reduces the toxicity of bactericidal materials in a normal physiological environment but also improves the therapeutic effect greatly. The relationship between the pre‐treatment defense system and targeted bactericidal system is an analogy to the concept of “*Yin‐Yang*” in Chinese culture, which is a complementary and mutually reinforcing relationship in the process of fighting clinical infections.

### 4.1 Pre‐Treatment Defense System

Implant infections can cause an imbalance in the immune system and surgical failure.^[^ ^123^ ^]^ In the past 20 years, the failure rate of prosthesis surgery is about 10%, and the revision rate of hip replacement surgery reaches 17.5%.^[^ ^116^ ^]^ Therefore, implant infection is a major problem that may inhibit bone integration of the implants and delay patients’ rehabilitation.^[^ ^124^ ^]^ Researchers have done a lot of meaningful works to avoid implant infections.^[^ ^125^ ^]^ For instance, antibiotics (e.g., gentamicin, carbenicillin, cephalothin, tobramycin, cefamandol, amoxicillin, and vancomycin) have been loaded on the implant's surface for resisting bacterial infections.^[^ ^126^ ^]^ But given the risk of subsequent antibiotic resistance, researchers have developed some good alternatives (e.g., chlorhexidine,^[^ ^127^ ^]^ poly(hexamethylenebiguanide^[^ ^128^ ^]^), chloroxylenol,^[^ ^129^ ^]^ and sliver^[^ ^130^ ^]^) for modifying the implant's surface. In addition, the nanostructures of the implant's surface also have been studied for reducing bacterial infections.^[^ ^131^ ^]^ Despite great advances in implant modification, the global infection rate during orthopedic surgery remains high (2–5%).^[^ ^132^ ^]^ The reasons why implants cannot resist bacterial infection include the following: 1) difficulty in maintaining long‐term antibacterial effect,^[^ ^133^ ^]^ 2) accumulation of bacterial fragments,^[^ ^134^ ^]^ and 3) failure to kill bacteria over time.^[^ ^122^, ^124^, ^135^ ^]^ Recently, a large number of works have been investigated to solve these problems, and are divided into three categories: 1) long‐term antifouling,^[^ ^16i^ ^]^ 2) smart self‐cleaning,^[^ ^16j^ ^]^ and 3) triggered bacterial killing.^[^ ^122^ ^]^ These strategies constitute to the pre‐treatment system for protecting implants from bacterial infection.

**Figure 5:**
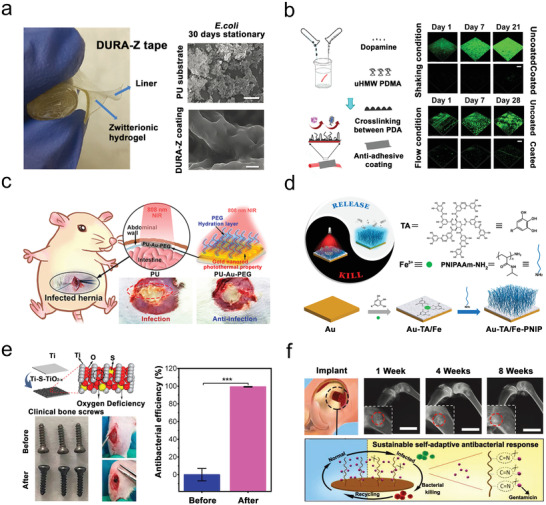
Pre‐treatment defense system via various strategies to avoid bacterial infections on implants. a,b) Strategy of long‐term anti‐fouling. a) DURA‐Z zwitterionic coating for long‐term antifouling up to 30 days. Reproduced with permission.^[^ ^16i^ ^]^ 2017, Wiley‐VCH. b) A nano‐durable PDA/PDMA for long‐term antifouling under flow/shaking conditions. Reproduced with permission.^[^ ^133c^ ^]^ Copyright 2018, American Chemical Society. c,d) Strategy of smart self‐cleaning. c) A PU‐Au‐PEG mesh with PTT and hydrophilic effect to clean bacteria for the treatment of hernia. Reproduced with permission.^[^ ^16j^ ^]^ Copyright 2020, American Chemical Society. d) A TA/Fe‐PNIP coating on Au surface to clean bacteria by thermal responsive size‐changing. Reproduced with permission.^[^ ^136^ ^]^ Copyright 2020, American Chemical Society. e,f) Strategy of triggered bacterial killing. e) An anoxic S‐doped TiO_2_ layer coated on Ti implant with almost 100% bactericidal effect triggered by ultrasound and NIR irradiation. Reproduced with permission.^[^ ^122^ ^]^ Copyright 2020, American Chemical Society. f) GS‐modified HA to release GS in acidic environment for the treatment of implant infections. Reproduced with permission.^[^ ^16h^ ^]^ Copyright 2019, Wiley‐VCH.

Currently, most of the antibacterial surface modifications are difficult to resist bacteria for a long period of time (>28 days) because it is difficult to maintain the stability of antibacterial coatings in complex environments in vivo.^[^ ^133b^ ^]^ A failed antibacterial surface may promote bacterial growth and results in bacterial drug resistance. In addition, the use of a large number of contaminated medical devices will make clinical treatment extremely difficult. Therefore, it is important to find a method to realize long term antifouling. Wang et al.^[^ ^16i^ ^]^ proposed a practical solution strategy, a super antifouling coating (DURA‐Z) formed by zwitterions (3‐((3‐acrylamidopropyl)‐dimethylammonio) propanoate) and commercial cyanoacrylate superglue. Under both shaking and stationary conditions with the high concentrations of bacteria or fungi (>10^9^ cells mL^−1^), the DURA‐Z coating could achieve almost “zero” bacterial attachment during a period of more than one month (**Figure** **5**a). In addition, the inter‐penetrating structure allowed the coating to adhere strongly to the material surface, which greatly improved the physical properties of shear performance, abrasion resistance, and compression resistance. Moreover, Mei et al.^[^ ^133c^ ^]^ utilized an ultra‐high molecular weight hydrophilic polymer (PDMA) to change the self‐assembly process of the polydopamine (PDA) on silicon wafers, to form a stable and long‐term anti‐adhesion nanocoating. Under both shock and flow conditions, this coating showed excellent antifouling and anti‐biofilm properties (>4 weeks) in the presence of >10^8^ CFU mL^–1^ bacteria (Figure 5b).

Leaving the dead bacteria on the surface not only poses risks to trigger inflammation and immune responses but also provides nutrients for bacterial growth.^[^ ^137^ ^]^ Therefore, in order to keep the medical device cleaning, the material surfaces should have the ability to remove any attached dead bacteria and its debris. A smart self‐cleaning modification strategy enables the materials to clean up bacterial debris and residual proteins effectively^[^ ^134b,c^ ^]^. For instance, Zhao et al.^[^ ^16j^ ^]^ developed a self‐cleaning polyurethane (PU) mesh via the combination of Au nanorods and PEG. PEG has inherent antifouling properties, which allowed the mesh to form superhydrophilic layer to prevent the accumulation of bacterial debris. Moreover, PU‐Au‐PEG displayed a good photothermal antibacterial property and showed a positive effect on the treatment of infected hernias in vivo (Figure 5c). Smart self‐cleaning material surfaces can also be constructed through functional switching under some stimuli (e.g., temperature,^[^ ^138^ ^]^ pH,^[^ ^139^ ^]^ wet,^[^ ^140^ ^]^ and light^[^ ^141^ ^]^). For example, Yu et al.^[^ ^142^ ^]^ proposed a self‐cleaning antibacterial surface by using a thermal‐responsive polymer (poly(*N*‐isopropylacrylamide, PNIPAM). Due to the presence of cationic biocide and nanopatterned PNIPAM brushes, nearly 81 ± 4% of attached bacteria were removed from the surface. Furthermore, Wang et al.^[^ ^136^ ^]^ grafted PNIPAM onto tannic acid (TA)/Fe^3+^ ion complex to form a self‐cleaning coating on Au substrate. This coating exhibited high photothermal bacteria‐killing efficiency (>99%) under the irradiation of NIR light, and thermal‐triggered self‐cleaning performance (removing >90% of the attached bacteria) (Figure 5d).

Developing responsive bactericidal surfaces on implants can avoid germicidal material leakage affecting normal tissues, maintain antibacterial properties, and kill bacteria over time so as to provide better cell attachment. For instance, Su et al.^[^ ^122^ ^]^ developed an anoxic S‐doped TiO_2_ layer that could endow the implant's surface with sonodynamic and photothermal ability. The antibacterial rate of the implant against *S. aureus* could reach 99.995% after 15 min of ultrasound and NIR light treatments (Figure 5e). Importantly, the structure and performance of the S‐doped titanium implant remained unchanged after immersion in water for 6 months, indicating that the internal modified titanium implant had a stable and excellent antibacterial performance under external stimulation in vivo.

In addition to cell death and inflammation caused by a bacterial infection, the competition between bacteria and cells is also a key factor affecting the success of implants. Killing bacteria and promoting the adhesion of cells to implants can help to improve the therapeutic effect. For instance, Jin et al^[^ ^16h^ ^]^ utilized gentamicin sulfate (GS) to functionalize hydroxyapatite (HA) surface through acid‐reactive bonds. The acidic environment created by bacterial metabolism could trigger the release of GS, and subsequently, induced a self‐adaptive antibacterial response. Moreover, the anti‐infective effect of HA‐GS in vivo was confirmed in a rabbit model with infected bone defects (Figure 5f). However, the degradation of the chemicals might have an unknown effect on the human body. Therefore, Zhang et al.^[^ ^135^ ^]^ developed TiO_2_ nanorod (diameter of 40–50 nm and length of about 1 µm) arrays with multiple synergetic antibacterial effects (ROS, hyperthermia, and bacterial puncturing). This design endowed implants with high bactericidal efficiency (99%) in vivo. Meanwhile, the TiO_2_ nanorod arrays could improve cell adhesion, proliferation, and osteogenic differentiation, further accelerating bone tissue regeneration.

### 4.2 Targeted Bactericidal System

When bacteria are transferred from the surface of medical devices to the human body, bacterial infections will cause dangerous diseases such as chronic wounds, bacterial bacteremia, and osteomyelitis.^[^ ^143^ ^]^ However, the complex physiological environment in the lesions of the wound and deep tissue makes treatment difficult.^[^ ^144^ ^]^ This requires antibacterial materials to kill bacteria completely under the intervention of the complex physiological environment. However, the design of traditional antibacterial materials tends to consider how to improve the sterilization efficiency without thinking about how to reduce the influence of the complex physiological environment on the materials.^[^ ^145^ ^]^ As a result, these antimicrobial materials are often highly toxic and have less help in treating infections. Therefore, it is necessary to develop advanced surface modification strategies to enhance the bactericidal effect at the location of the lesion, thereby reducing the interaction between the material and the normal physiological environment.^[^ ^87b^ ^]^ The method that endows antibacterial material with bacterial capturing and targeting properties, is a promising strategy to achieve efficient and accurate attacks on bacteria, in chronic wounds and deep tissue/organs.

Chronic wounds are common in patients suffering from diabetes, vascular disease, aging, as well as hemoglobinopathies. Treatment of infected wounds tends to become difficult due to the presence of large amounts of tissue fluid, healthy cells, necrotic cells, and bacteria.^[^ ^146^ ^]^ The targeted bactericidal system may allow the materials to capture bacteria and release germicidal substances at very close proximity, thus reducing the native effect of bactericidal substances on normal physiological tissues. Targeted bactericidal system can capture bacteria at the chronic wounds by electrostatic attraction,^[^ ^87b^ ^]^ receptor‐ligand interactions,^[^ ^147^ ^]^ covalent bonding,^[^ ^145^ ^]^ and bacterial trapping.^[^ ^148^ ^]^

**Figure 6:**
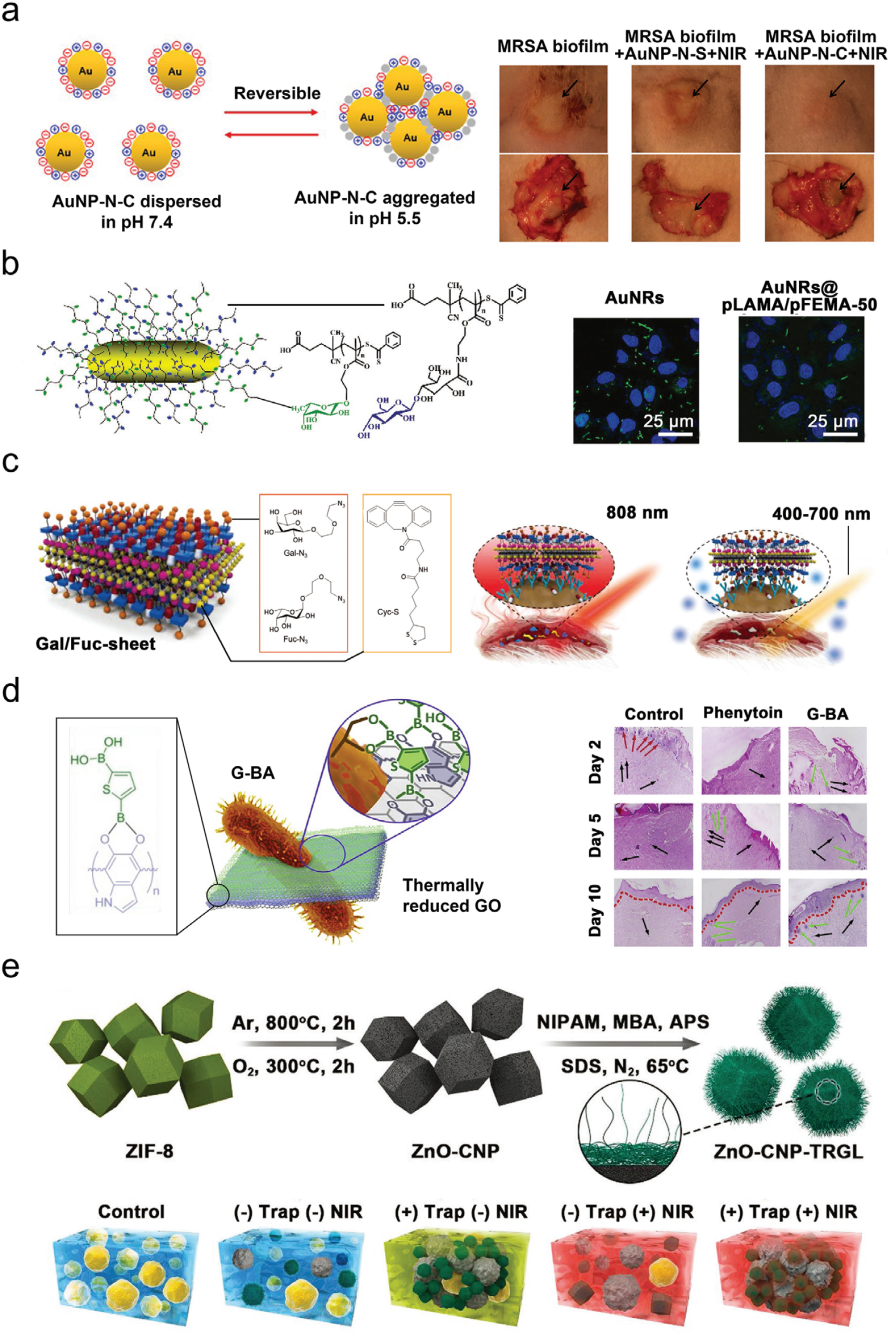
Targeted bactericidal system via various strategies for capturing bacteria in chronic wounds. a) Strategy of electrostatic attraction. pH‐triggered charge‐reversible AuNP to capture MASR in an acidic environment for abscesses therapy. Reproduced with permission.^[^ ^87b^ ^]^ Copyright 2017, American Chemical Society. b,c) Strategy of receptor‐ligand interactions. b) Gal/Fuc‐coated AuNPs with dual‐targeting ability to capture *P. aeruginosa*, without cytotoxicity. Reproduced with permission.^[^ ^149^ ^]^ Copyright 2018, Wiley‐VCH. c) Light responsible Gal/Fuc‐sheet loaded with antibiotics to treat multidrug‐resistant *P. aeruginosa* infections. Reproduced with permission.^[^ ^147a^ ^]^ Copyright 2019, Wiley‐VCH. d) Strategy of covalent bonding. G‐B(OH)_2_ covalently binding to the membrane of *E.coli* for curing wound infections. Reproduced with permission.^[^ ^145^ ^]^ Copyright 2019, Elsevier Ltd. e) Strategy of bacterial trapping. ZnO‐CNP‐TRGL with size switchable ability to trap bacteria under NIR irradiation. Reproduced with permission.^[^ ^148^ ^]^ Copyright 2019, Wiley‐VCH.

Due to the electronegativity of the bacterial surface, the cationic molecules can be adsorbed to the bacteria by electrostatic attraction.^[^ ^150^ ^]^ Developing charge switchable surfaces is an effective strategy to capture bacteria and reduce cytotoxicity to normal cells.^[^ ^150^ ^]^ For instance, Yu et al.^[^ ^151^ ^]^ reported a graphene nanosheet coated with glycol chitosan (GCS‐PDA@CG), a water‐soluble chitosan derivative with stealth properties and a pH‐sensitive switchable charge, to target bacteria in chronic wounds. GCS‐PDA@CG could kill 99% bacteria in an infected wound after irradiation of 808 nm laser for 8 min. In addition, the charge switching of the material surface can be achieved by the strong and weak electrolytes. For example, Hu et al. reported a bacterial targeting Au nanoparticle modified by weak electrolytic 11‐mercaptoundecanoic acid (HS‐C_10_‐COOH) and strong electrolytic (10‐mercaptodecyl) trimethylammonium bromide (HS‐C_10_‐N4).^[^ ^87b^ ^]^ The mixed charged monolayers on the surface of AuNPs illustrated a quick pH‐dependent charge conversion, allowing AuNPs to be well‐dispersed in the healthy tissues whilst adhering strongly to the weakly acidic MRSA biofilms (pH ≈ 5.5). Compared with the dispersive AuNPs, the aggregated AuNPs showed better photothermal bactericidal effect (81%) in the subcutaneous abscess, without any side effects on normal tissue under NIR light irradiation (**Figure** **6**a).

Besides the electrostatic attraction mentioned above, receptor‐ligand interactions are also an effective strategy to target characteristic molecules in the bacterial cell wall. Glycans derivatives (e.g., the mannose, fucose, and monosaccharides galactose) can specifically bind to lectin^[^ ^152^ ^]^ (a carbohydrate‐binding protein on the surface of bacteria), which has been used widely in antibacterial material modification to target bacteria.^[^ ^153^ ^]^ For example, Guo et al.^[^ ^154^ ^]^ reported that galactose‐modified black phosphorus nanosheet could improve the affinity to bacteria, which killed *P. aeruginosa* totally at low concentrations (8 µg mL^–1^). Moreover, the strength of this affinity could be improved by the adjustment of the number and species of glycosyl groups on the surface of the materials. For instance, Zhao et al.^[^ ^149^ ^]^ developed Au nanoparticles (AuNPs) coated with galactose (Gal) and fucose (Fuc) ligands to target two different lectins (LecA and LecB) on *P. aeruginosa* (Figure 6b). This strategy endows AuNPs with lower effective antibacterial concentration (400 µg mL^–1^), faster adhesion rate (taking 50 min to reach 50% adhesion capacity), and higher sterilization efficiency (killing nearly 100% bacteria within 3 min under the NIR irradiation) than other materials reported previously.^[^ ^155^ ^]^ The targeting effect of glycans derivatives can also be combined with drug release strategies. For instance, Hu et al.^[^ ^147a^ ^]^ reported a multifunctional glycol‐sheet (Gal/Fuc‐sheet) loaded antibiotics (ceftazidime) allowing for double light‐triggered therapy to treat multidrug‐resistant *P. aeruginosa* infections (Figure 6c). Gal/Fuc‐sheet could achieve the thermal release of antibiotics and ROS production under irradiation of NIR (808 nm) light and white light (400–700 nm), respectively, and cure the infected wounds within 4 days.

In addition to glycans derivatives, boronic acid (BA) is often used as a targeting group that can bind specifically to lipopolysaccharide (LPS, widely present on the cell walls of bacteria).^[^ ^156^ ^]^ For example, Beyranvand et al.^[^ ^145^ ^]^ developed functional BA‐coated graphene (G‐B(OH)_2_), which could inhibit the growth of *E. coli* and *B. cereus* at low concentrations (29.5 and 38.7 µg mL^–1^, respectively), and cured the diabetic wound in 10 days (Figure 6d). The targeting effect of BA could also be combined with PDT strategies. For example, Galstyan et al. functionalized the surface of silicon phthalocyanine with BA, which exhibited high bacterial killing efficiency at low concentrations (MIC: ≈10 µm), and destroyed biofilms of gram‐negative *E. coli* effectively (reducing 50% within 30 min) under NIR light.^[^ ^157^ ^]^ In addition to PDT, BA can also be combined with other bactericidal strategies. For example, Wang et al.^[^ ^158^ ^]^ prepared a multifunctional chip coated with 4‐mercaptophenyl boronic acid and silver nanoparticles, with a good capture efficiency (≈60%) and high antibacterial rate (≈97%) of the pathogenic bacteria.

Trapping bacteria by the size transformation of antibacterial material is also another interesting strategy for treatment of chronic wounds. The well‐dispersed nanoparticles can form micrometer aggregations to trap bacteria by coating with thermo‐responsive gel. For example, Yang et al.^[^ ^148^ ^]^ reported a bactericidal ZIF‐derived nanocarbon coated with PNIPAM (ZnO‐CNP‐TRGL) to achieve size transformation for bacterial trapping (Figure 6e). This nanocarbon showed high photo‐to‐thermal conversion efficiency (the temperature could increase from 23.5 to 55 °C within 5 min), and a rapid size transformation from nanodispersions (<100 nm) to micrometer (>2 µm) aggregations upon NIR irradiation, releasing localized massive heat and abundant Zn^2+^ ions, for the direct disruption of the bacterial membrane and intracellular proteins, resulting in a nearly 100% bacterial killing ratio. Moreover, trapping bacteria can also be achieved by micro‐morphology to avoid germicidal damage to normal cells. For instance, Hu et al.^[^ ^159^ ^]^ reported a wound dressing composed of biomimetic microtopography (mimicking the feature of shark‐skin), PNIPAM gel, and Au nanostars. This strategy involved a critical dimension for a surface feature (3 µm), which was larger than a single bacterium (normally 0.2–2.0 µm in diameter) but smaller than a single mammalian cell (normally larger than 5 µm). This microtopography allowed the thermo‐responsive gel to shrink toward the surface, which kept Au nanostars producing localized heat at a safe distance from the host cells, thereby killing *S. aureus* (≈95%) that were trapped within the gaps effectively. As a result, infected wounds were cured within 6 days.

In addition to chronic wounds, another serious clinical infection problem is that bacterial infections can occur in deep tissue and/or organ, causing diseases such as bacteremia, sepsis, endocarditis, and osteomyelitis.^[^ ^160^ ^]^ The conventional treatment strategy is usually intravenous antibiotics,^[^ ^161^ ^]^ with the following disadvantages: 1) low drug enrichment; 2) disruption of intestinal flora; and 3) accelerated bacterial resistance.^[^ ^161^ ^]^ In order to avoid over‐usage of antibiotics and to achieve more effective treatment of bacterial infection, antibacterial materials need to possess excellent penetration and retention abilities in the infected site. The targeted bactericidal system may work to solve the problem as mentioned above by various strategies such as surface charging switching,^[^ ^16k^ ^]^ bionics modification,^[^ ^162^ ^]^ and application of micro/nanorobots.^[^ ^163^ ^]^

**Figure 7:**
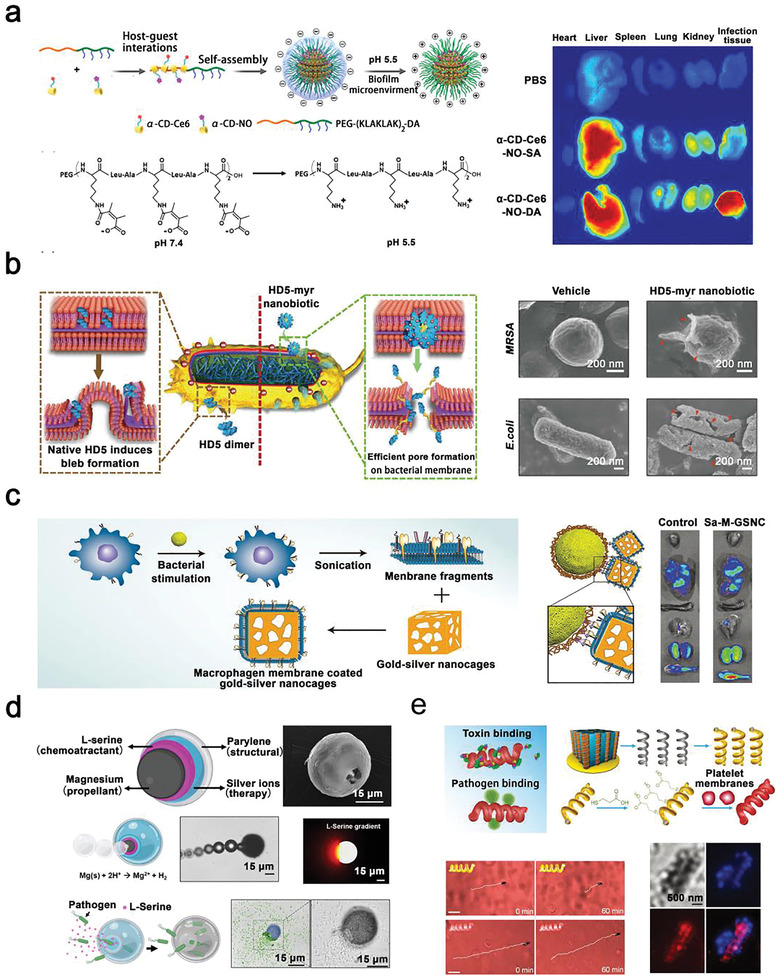
Targeted bactericidal system via various strategies for targeting bacteria in deep tissue or organs. a) Strategy of surface charging switching. *α*‐CD‐Ce6‐NO‐DA nanoparticles with pH‐triggered switchable charge surface to target biofilm (pH ≈ 5.5) in deep infection tissue. Reproduced with permission.^[^ ^16k^ ^]^ Copyright 2020, American Chemical Society. b,c) Strategy of bionics modification. b) C‐terminally myristoylated HD5 nanobiotic inspired by HDP to destroy bacterial membrane. Reproduced with permission.^[^ ^162^ ^]^ Copyright 2018, American Chemical Society. c) A macrophage membrane‐coated GSNC to target *S. aureus* infected tissue. Reproduced with permission.^[^ ^164^ ^]^ Copyright 2018, Wiley‐VCH. d,e) Strategy of micro/nanorobots. d) An onion‐like microrobot for capturing and killing bacteria automatically. Reproduced with permission^[^ ^165^ ^]^ Copyright 2019, Wiley‐VCH Verlag GmbH & Co. KGaA. e) A PL‐motors for the isolation of bacterial toxin in blood. Reproduced with permission.^[^ ^163^ ^]^ Copyright 2017, Wiley‐VCH.

Surface charge switching is an effective strategy to endow antibacterial materials with long‐term circulation in the blood and effective penetration at an infected site.^[^ ^16k^ ^]^ Hu et al.^[^ ^16k^ ^]^ reported a surface charge switchable supramolecular nanoparticle (*α*‐CD‐Ce6‐NO‐DA). The acid‐triggered charge reversal property benefited the long‐term circulation of *α*‐CD‐Ce6‐NO‐DA in the bloodstream (pH 7.4). In vitro fluorescence images demonstrated that *α*‐CD‐Ce6‐NO‐DA had excellent penetration and retention abilities in the biofilm infected sites (pH 5.5) after injection for 24 h. Upon permeation into the biofilm, these surface charge switchable nanoparticles could release an abundance of nitric oxide, reactive nitrogen species, and reactive oxygen species under irradiation of 660 nm laser, thus resulting in 100% bacterial killing (**Figure** **7**A).

In addition to charge switching, bionics modification is another useful strategy for bacterial targeting in the infected tissue. In this paradigm, the antibacterial materials can be modified with antimicrobial human defensin peptide,^[^ ^166^ ^]^ antibodies,^[^ ^167^ ^]^ and cell membranes,^[^ ^168^ ^]^ to obtain long‐term circulation and efficient bacterial targeting ability. Host defense peptides can endow the material with bacterial targeting ability and excellent biocompatibility.^[^ ^162^ ^]^ For instance, a self‐assembled nanoparticle from C‐terminally myristoylated human alpha‐defensin 5, could destroy the cell membrane of the bacteria and, at the same time, selectively kill *E. coli* and MRSA completely with 12.5 µg mL^–1^ (Figure 7B).^[^ ^162^ ^]^ Inspired by a human defensin‐6 antimicrobial peptide, Fan et al.^[^ ^150b^ ^]^ also reported a self‐assembling nanoparticle with a special peptide sequence (KLVFF‐RLYLRIGRR). The RLYLRIGRR peptide sequence allowed self‐assembly nanoparticle target lipoteichoic acid on the surface of gram‐positive bacteria specifically. After binding to bacteria, the KLVFF peptide skeleton allowed nanoparticles to form a nanofibers network through *π*‐*π* interactions to capture bacteria. Notably, this nanoparticle (200 µm) can achieve a 100% cure rate in mice with MARS bacteremia due to its ability of limiting bacterial movement and division in blood.

Another effective bionics strategy is coating bactericidal material with cell (such as macrophage,^[^ ^169^ ^]^ leukocyte,^[^ ^170^ ^]^ platelet,^[^ ^163^ ^]^ red blood cell,^[^ ^171^ ^]^ and bacteria^[^ ^168^ ^]^) membranes. Due to the presence of various receptor proteins,^[^ ^163^ ^]^ cell membranes can bind to bacterial molecular patterns, thus improving the retention ability of antibacterial materials in the infected site.^[^ ^169^ ^]^ In particular, macrophages can express various Toll‐like receptors (TLR), in which TLR2 and TLR4 can identify the teichoic acid of Gram‐positive bacteria and lipopolysaccharide of Gram‐negative bacteria, respectively.^[^ ^172^ ^]^ This property enables macrophage membranes have attracted researchers' attention as a kind of bacterial targeting material.^[^ ^173^ ^]^ For example, Wang et al.^[^ ^164^ ^]^ reported a gold and silver nanocage (GSNC) coated with macrophage membrane by extrusion technique. Due to the bacterial recognizing receptors on macrophage membranes, macrophage membranes‐coated GSNC could be effectively enriched in the infection site. Moreover, those functionalized GSNC had a strong affinity to *S. aureus* infection site as observed by fluorescence imaging (Figure 7C).

The application of micro/nanorobot is another promising antibacterial strategy,^[^ ^174^ ^]^ because they have an efficient capacity for controllable locomotion through exogenous dynamics^[^ ^175^ ^]^ (e.g., magnetic, ultrasound, and light energy propulsion) or endogenous dynamics (e.g., chemical or biological reactions).^[^ ^176^ ^]^ Surface modification can further help micro/nanorobots to destroy bacteria or biofilms easily on difficult‐to‐access areas such as implants, catheters, teeth, and mucosal.^[^ ^177^ ^]^ For instance, Fernando et al.^[^ ^165^ ^]^ developed an onion‐like microrobot composed of an Mg sphere and multiple functional transient layers (l‐serine chemoattractant and silver ions). Mg sphere acted as an engine and could react with acid to produce hydrogen bubbles, which in turn drives the robot to the infection site. When the Mg sphere was totally depleted by gastric acid, chemoattractant l‐serine will be released to induce bacterial aggregation for capturing and killing the bacteria with high efficiency (94%) (Figure 7D). Furthermore, Li et al.^[^ ^163^ ^]^ developed a magnetic helical nanorobot coated with platelet‐membrane (PL‐motors) for the adsorption and isolation of platelet‐targeted bacterial toxin. The helical shape allowed PL‐motors to rotate forward since it was driven by a magnetic field. Meanwhile, the natural platelet cell membranes coating on the surface made the PL‐motors moved smoothly and adsorbed many toxic factors and pathogenic microorganisms in the blood, thereby increasing cell viability (92%) by four times as compared to the control group (21%) (Figure 7E).

## 5 Summary and Perspectives

In this review, we summarize the key factors on material surfaces that influence bacteria from adhesion behavior to biofilm formation and, thereafter, provide a comprehensive discussion on various surface modification strategies to guide designs against bacterial adhesion and biofilms formation. Furthermore, we review the emerging strategies to develop antibacterial materials with clinical potential and present them as a “super antibacterial system” with the necessary multifunctional abilities such as high‐efficiency antibacterial effects, long‐term effects, bacterial targeting, and biocompatibility. It should be noted that some antibacterial strategies are still in their early stage of pre‐clinical development. For instance, surface patterning technology has been an important strategy in the antibacterial field. But it is still in its early development where there are many issues to overcome. Some cases we discussed tend to expensive and cost‐ineffective from a fundamental standpoint. But, these cases are frontier researches in this field and they do provide a kind of novel strategy regarding surface modification to avoid bacterial infection, which is potential of great significance. Summarizing these advanced examples together can contribute to the better development of antimicrobial materials.

Nowadays, a large number of useful antimicrobial surfaces have been reported to promote the development of clinical antimicrobial materials. Among these studies, antimicrobial modifications applied to surface of the implant have attached the most attention because of a high incidence of the implant‐associated bacterial infections.^[^ ^178^ ^]^ Methods for constructing the antimicrobial surface of implants can be divided into three categories:^[^ ^179^ ^]^ 1) surface properties of the implants, such as hydrophilicity, potential energy, porosity, and roughness were changed to affect the colonization of bacteria on implant surface; 2) antibacterial factors were blended with the raw materials to construct the implants with the antibacterial surface; 3) antibacterial substance is fixed on the surface of the implant to form a uniform coating, through chemical grafting, plasma spraying, layer‐by‐layer self‐assembly, electrophoresis, and other ways. In addition, the use of antimicrobial surfaces in hydrogels is also of great interest, as hydrogel is considered as an ideal wound dressing.^[^ ^180^ ^]^ Antibacterial hydrogels can be roughly divided into two kinds:^[^ ^181^ ^]^ 1) hydrogels containing antimicrobial agents; and 2) hydrogels with inherent antibacterial ability.

Although antibacterial materials have progressed rapidly, the advancement of the clinical application can only become possible after addressing several significant issues. The first issue is to design new antibacterial strategies, which require a deeper understanding of the bacterial structures.^[^ ^182^ ^]^ New antimicrobial strategies can provide new opportunities for clinical therapy and avoid the development of bacterial resistance greatly. Second, biocompatibility must be considered carefully during the development of antibacterial materials.^[^ ^183^ ^]^ Although no obvious toxicity in vivo is claimed for the materials in many studies, long‐term investigation on in vivo toxicity is still lacking. Finally, it is necessary to establish a standardized quality evaluation system.^[^ ^184^ ^]^ The antibacterial performance varies greatly for the developed materials in various studies, which is a major obstacle to the development of the clinical application. An advanced evaluation system can help researchers evaluate antibacterial materials objectively and screen out promising material design effectively.

At present, efforts are devoted to developing antimicrobial materials that can have real potential for clinical application, among which major focuses and interests are on providing solutions for the foot ulcer in diabetic patients, antibacterial medical implants, and the treatment of bacteremia.^[^ ^185^ ^]^ We believe that bacterial responsiveness and targeting capabilities are promising for the design of antimicrobial materials such as nanoparticles with bacterial targeting,^[^ ^186^ ^]^ antibacterial micro–nano robots,^[^ ^187^ ^]^ and smart hydrogel materials.^[^ ^188^ ^]^ These antibacterial materials are promising for the treatment of clinical infections: 1) Targeting materials with bacterial‐related selectivity can largely avoid the bactericidal effects of antibacterial materials interfering with normal tissue cells.^[^ ^186^ ^]^ Most of these targets are based on the inherent structure of bacteria, which makes it difficult for the bacteria to develop drug resistance.^[^ ^189^ ^]^ At the same time, targeting can enhance the affinity between bacteria and materials, which could achieve different antibacterial patterns such as active adsorption on bacteria and limiting bacterial migration;^[^ ^190^ ^]^ 2) Unique physical forms (spiral, star, and spherical) of micro/nano robots endow them with a strong ability to tear biofilm.^[^ ^187^ ^]^ The vast majority of micro–nano robots can be manipulated artificially to achieve long‐distance targeting for infected sites, which can be helpful for removing bacteria effectively for various medical equipment and implants.^[^ ^191^ ^]^ Moreover, the surface of micro/nanorobots can be modified with various coating to endow the robots with multifunction such as drug delivery, blood cleaning, and unique movement patterns;^[^ ^192^ ^]^ 3) Smart hydrogels that obtain usage widely in drug carrier, tissue engineering, and wearable smart devices, are characterized with unique rich water content, 3D network structure, and various molecular designs.^[^ ^188^ ^]^ These smart‐hydrogels can achieve specific responses to the various micro‐environment in the treatment of diabetic foot ulcers causing high amputation rates.^[^ ^193^ ^]^ Through the functionalization design of a 3D network, the construction of multifunctional intelligent hydrogel can be realized to treat diabetic foot ulcers from many aspects such as antibacterial, wound cleaning, and insulin‐responsive release.^[^ ^194^ ^]^

Moreover, surface design is the best way to create antimicrobial materials to avoid the spread of microorganisms such as virus which are mainly transmitted through contaminated surfaces and exhaled droplets.^[^ ^195^ ^]^ Recently, the COVID‐19 pandemic has caused an increased demand for antimicrobial treatments that can keep surfaces clean, particularly in health care settings.^[^ ^196^ ^]^ Some of the design strategies discussed in this review can be used to destroying or inhibiting the spread of virus. For instance, cationic polymeric membranes such as polyethyleneimine can achieve an excellent killing effect on enveloped viruses and a good filtering effect on non‐enveloped waterborne viruses in water treatment.^[^ ^197^ ^]^ In addition, Heat, oxidative species (e.g., •OH and O_2_−), and metal ions (e.g., silver and copper) can cause virus death by inducing protein denaturation and DNA/RNA damage.^[^ ^198^ ^]^ Nowadays, there are a lot of literatures related to antibacterial materials, but relatively few literatures related to antiviral materials. To develop more effective antiviral materials and realize the combination of antiviral and antibacterial strategies can mitigate the spread of pathogenic microorganism.

Continuous breakthroughs in the field of materials science and the expansion of antimicrobial molecular libraries will certainly enrich strategies for the design and development of antimicrobial material with clinical potential. Advanced strategies that integrate bacterial biology and advanced material designs will develop materials with desirable antimicrobial performances and, in turn, provides excellent solutions for the rapid development of modern human healthcare.

## Conflict of Interest

The authors declare no conflict of interest.
